# Molecular cytogenetic identification of a novel wheat-*Agropyron elongatum* chromosome translocation line with powdery mildew resistance

**DOI:** 10.1371/journal.pone.0184462

**Published:** 2017-09-08

**Authors:** Xiaojun Li, Xiaoling Jiang, Xiangdong Chen, Jie Song, Cuicui Ren, Yajuan Xiao, Xiaohui Gao, Zhengang Ru

**Affiliations:** College of Life Science and Technology, Collaborative Innovation Center of Modern Biological Breeding, Henan Province, Henan Institute of Science and Technology, Xinxiang, Henan, China; Institute of Genetics and Developmental Biology Chinese Academy of Sciences, CHINA

## Abstract

*Agropyron elongatum* (Host.) Neviski (synonym, *Thinopyrum ponticum* Podp., 2n = 70) has been used extensively as a valuable source for wheat breeding. Numerous chromosome fragments containing valuable genes have been successfully translocated into wheat from *A*. *elongatum*. However, reports on the transfer of powdery mildew resistance from *A*. *elongatum* to wheat are rare. In this study, a novel wheat-*A*. *elongatum* translocation line, 11-20-1, developed and selected from the progenies of a sequential cross between wheat varieties (Lankaoaizaoba, Keyu 818 and BainongAK 58) and *A*. *elongatum*, was evaluated for disease resistance and characterized using molecular cytogenetic methods. Cytological observations indicated that 11-20-1 had 42 chromosomes and formed 21 bivalents at meiotic metaphase I. Genomic *in situ* hybridization analysis using whole genomic DNA from *A*. *elongatum* as a probe showed that the short arms of a pair of wheat chromosomes were replaced by a pair of *A*. *elongatum* chromosome arms. Fluorescence *in situ* hybridization, using wheat D chromosome specific sequence pAs1 as a probe, suggested that the replaced chromosome arms of 11-20-1 were 5DS. This was further confirmed by wheat SSR markers specific for 5DS. EST-SSR and EST-STS multiple loci markers confirmed that the introduced *A*. *elongatum* chromosome arms belonged to homoeologous group 5. Therefore, it was deduced that 11-20-1 was a wheat-*A*. *elongatum* T5DL∙5AgS translocation line. Both resistance observation and molecular marker analyses using two specific markers (BE443538 and CD452608) of *A*. *elongatum* in a F_2_ population from a cross between line 11-20-1 and a susceptible cultivar Yannong 19 verified that the *A*. *elongatum* chromosomes were responsible for the powdery mildew resistance. This work suggests that 11-20-1 likely contains a novel resistance gene against powdery mildew. We expect this line to be useful for the genetic improvement of wheat.

## Introduction

Wheat (*Triticum aestivum* L., 2n = 6x = 42, AABBDD) is one of the most important cereals worldwide. Genetic erosion has narrowed the genetic base of common wheat as a result of high selection pressure and repeated use of the adapted germplasm [1]. The wild relatives and related species of wheat represent a large reservoir of useful traits, e.g., good quality, tolerance to low-temperature and salt, resistance to wheat disease and superior numbers of florets and grains, and may provide important genetic resources for broadening the genetic diversity of wheat [2].

*Agropyron elongatum* (Host.) Neviski (synonym, *Thinopyrum ponticum* Podp., 2n = 70), a species closely related to wheat, has been used extensively as a valuable source of genes for wheat breeding. Genes that confer exceptional traits include resistance to leaf rust [3], stem rust [4], stripe rust [5], *Fusarium* head blight [6], common root rot [7], tan spot [8], and dwarfism [9] as well as increased photosynthetic efficiency [10], increased yield and biomas [11]. These traits have been introduced into wheat successfully from *A*. *elongatum*, and a few useful wheat cultivars or lines have been developed, such as Xiaoyan 6, Xiaoyan 759, Xiaoyan 22, Gaoyou 504 and Shanrong 3 in China [5], and Oasis and Seri in Mexico [11], indicating *A*. *elongatum* can play an important role in the improvement of wheat.

Powdery mildew of wheat caused by *Blumeria graminis* f. sp. *tritici* (*Bgt*) is one of the major diseases all over the world, and it can cause yield losses up to 50% in extremely susceptible cultivars [12]. In recent years, powdery mildew severely threatened wheat production in China. For example, the area of powdery mildew in wheat comes up to 7,400,000 hectares in 2016 in the main wheat-producing regions of China [13]. To date, approximately 42 loci with more than 70 alleles conferring resistance to powdery mildew have been reported [14]. However, many of the available resistance genes have been overcome by newly emerged virulent isolates and can not meet the needs of wheat resistance breeding. Breeding resistant cultivars is generally accepted to be the most economical and effective means to control the disease. Hence, there is an urgent need to exploit new powdery mildew resistant genes for wheat breeding.

Genomic *in situ* hybridization (GISH) can easily identify the presence of alien chromosomes or introgression segments that have been introduced into wheat. Fluorescence *in situ* hybridization (FISH) is an efficient and accurate method to discriminate constitution of chromosomes through localizing highly repetitive DNA sequences to specific chromosome sites [15]. In addition to cytological methods, with the development of molecular biology, diverse molecular markers, such as simple sequence repeat (SSR), random amplified polymorphic DNA (RAPD), sequence-characterized amplified region (SCAR), sequence-tagged site (STS), and PCR-based landmark unique gene (PLUG) [16], have become extremely useful in identifying alien chromatin that has been integrated into the wheat genome and investigating genomic relationships between wheat and its relatives.

In this study, we identified a novel wheat-*A*. *elongatum* translocation line 11-20-1 using molecular cytogenetic methods. In addition, the resistance to powdery mildew of 11-20-1 was evaluated. We predict that the validations provided in this paper will facilitate the utilization of this line in wheat breeding programs.

## Materials and methods

### Plant materials

The materials included wheat cultivars Chinese Spring (CS), Lankaoaizaoba, Keyu 818 and BainongAK 58 (2n = 6x = 42, AABBDD), *A*. *elongatum*, two nulli-tetrasomic lines based on CS (CSN5DT5A and CSN5AT5D), and a wheat-*A*. *elongatum* derivate line 11-20-1. In our group, a F_1_ plant from a cross between *A*. *elongatum* introduced from Australia and bread wheat Lankaoaizaoba was provided kindly by Mr Tianming Shen. It was further crossed with wheat line Keyu 818, and backcrossed two times using wheat cultivar BainongAK 58, and then selfed for two generations. Finally, we selected line 11-20-1 based on its powdery mildew resistance under natural conditions and identical agronomical traits. Chinese Spring genomic DNA was used as a blocker in the GISH and FISH analyses and as control in molecular marker analyses. A F_2_ population derived from the cross between line 11-20-1 and a powdery mildew high susceptible cultivar Yannong 19 was also used in this study.

### Chromosome preparation

Seeds were germinated on moistened filter paper in petri dishes maintained in a growth chamber at 25 *°*C in the dark. Root tips were collected and placed immediately in ice water for 20–24 h, fixed in ethanol-acetic acid (3:1) fixative for 2 d and stored in 70% (v/v) ethanol. Root tips were squashed in 45% (v/v) acetic acid. The slides with mitotic metaphase cells were frozen in liquid nitrogen, and they were stored at -20°C until use after cover slips were removed. When plants reached the flag leaf stage, spikes were fixed in ethanol-acetic acid-chloroform (6:1:3) fixative for 12 h and stored in 70% (v/v) ethanol. The anthers were squashed in 45% (v/v) acetic acid to examine the meiotic chromosomes.

### GISH and FISH analysis

GISH analysis was conducted to detect *A*. *elongatum* chromatin in 11-20-1. Genomic DNA of *A*. *elongatum* and Chinese Spring wheat was extracted separately using the method reported by Sharp et al. [17]. Whole genomic DNA of *A*. *elongatum* was labeled with the Digoxigenin (DIG)-Nick Translation Mix (Roche, Mannheim, Germany) and used as a probe. The genomic DNA from Chinese Spring wheat was sheared for 5 min by ultrasonication and used as a blocker. The hybridization mixture was added onto the slides and denatured at 80 *°*C for 6 min. Chromosome denaturation, hybridization, and hybridization signal detection were carried out as described by Han et al. [18]. The cells with good hybridization signals were viewed and photographed (Olympus BX51, Japan) equipped with a CCD camera.

The highly repeated DNA sequence, pAs1, was labeled with the DIG-Nick Translation Mix, and hybridization and the detection were the same as in the GISH protocol. This pAs1 probe contains a 1-kb repetitive DNA sequence from *Aegilops tauschii* and enables identification of chromosome 1A and all the D-genome chromosomes of wheat [19].

### Molecular marker analysis

DNA was extracted from leaves of 11-20-1 and its parents and two nulli-tetrasomic lines (CSN5DT5A and CSN5AT5D) by using the same method as above. Here, 560 SSR markers located on different chromosomes, including Xgwm, Xwmc, Xbarc, Xcfa and Xcfd sources (http://wheat.pw.usda.gov/cgi-bin/graingenes/browse.cgi), were used to characterize the genetic composition of 11-20-1. Chinese Spring, Lankaoaizaoba, Keyu 818, BainongAK 58, *A*. *elongatum*, and CSN5DT5A and CSN5AT5D were used as control.

To determine the homoeologous group relationships of the introduced *A*. *elongatum* chromosomes in 11-20-1, we used 390 EST-SSR (Xcfe, Swes and Xcwem), 145 EST-STS (http://wheat.pw.usda.gov/SNP/new/pcr_primers.shtml) and 149 PCR-based landmark unique gene (PLUG) markers [16] from seven homoeologous groups of wheat chromosomes.

PCR was performed as described by Li et al. [20] using a PTC-200 thermocycler (MJ Research, Watertown, MA). The cycling parameters were, 94 *°*C for 4 min to pre-denature; followed by 36 cycles of 94 *°*C for 1 min, 55~6 0 *°*C for 1 min, and 72 *°*C for 1 min (SSR and EST-SSR amplification) or 2 min (EST-STS and PLUG amplification); and then a final extension at 72 *°*C for 8 min. PCR products were separated on 8% polyacrylamide non-denatured gels and were visualized following silver-staining.

The marker, pLeUCD2 specific for E genome [21,22], was used to detect E chromatin in 11-20-1 and the primer sequences were as follows: F: 5′-ACAATCTGAAAATCTGGACA-3′, R: 5′-TCATATTGAGACTCCTATAA-3′). Amplification was carried out following a program at 94 *°*C for 4 min; 94 *°*C for 1 min; 50 *°*C for 1 min; and 72 *°*C for 1 min for 36 cycles. The PCR products were separated on 8% polyacrylamide non-denatured gels.

### Powdery mildew assessment

Powdery mildew resistance of 11-20-1 at the seedling and adult plant stages was evaluated under natural conditions using the wheat parents (Lankaoaizaoba, Keyu 818 and BainongAK 58) and CS as control in greenhouses that have favorable environments for powdery mildew development in Henan Institute of Science and Technology, Xinxiang, China. At the seedling stage, ten seeds per pot for each line were used to evaluate resistance to powdery mildew with two replications. At the one-to two-leaf stage, seedlings were inoculated with a mixture of Bgt collected from other susceptible varieties. Infection types (IT) on individual plants were evaluated about two weeks after inoculation when the control variety CS were all fully infected. A 0–4 infection scale was used to describe IT scores [23], as follows: 0, no visible symptoms; 0; necrotic flecks without sporulation, and 1–4 for strongly resistant, resistant, susceptible and strongly susceptible, respectively. Adult plant powdery mildew test was also carried out in greenhouses in several growing season. The materials were grown in two 2-m long and three row-plot trials. IT on individual plants were recorded based on the same scale described as above when the flag leaves of the susceptible control were fully expanded.

## Results

### Cytological identification

**Fig 1:**
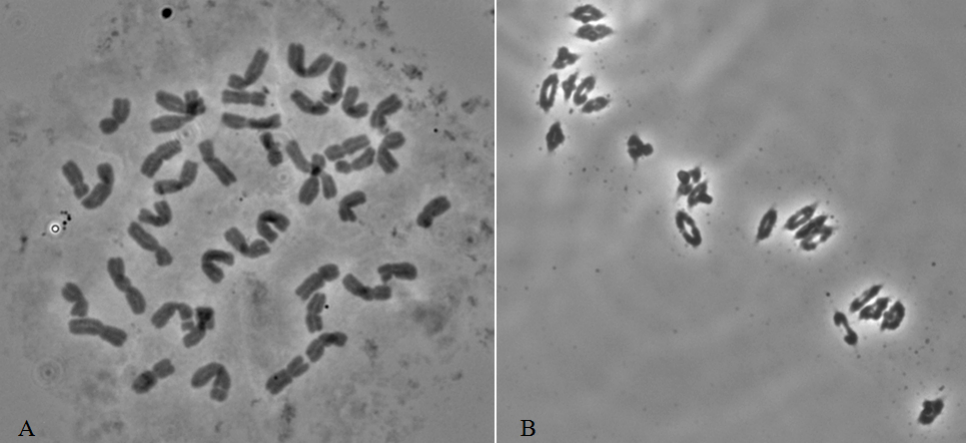
Mitotic (a) and meiotic I (b) chromosome characteristics of 11-20-1, (a) 2n = 42, (b) 2n = 21II.

Root-tip chromosome counts and meiotic observations of pollen mother cells in ten plants showed that 11-20-1 had a mitotic chromosome number of 2n = 42 (Fig 1A) and formed 21 bivalents at meiotic metaphase I (Fig 1B), indicating highly cytological stability.

### GISH and FISH analysis

**Fig 2:**
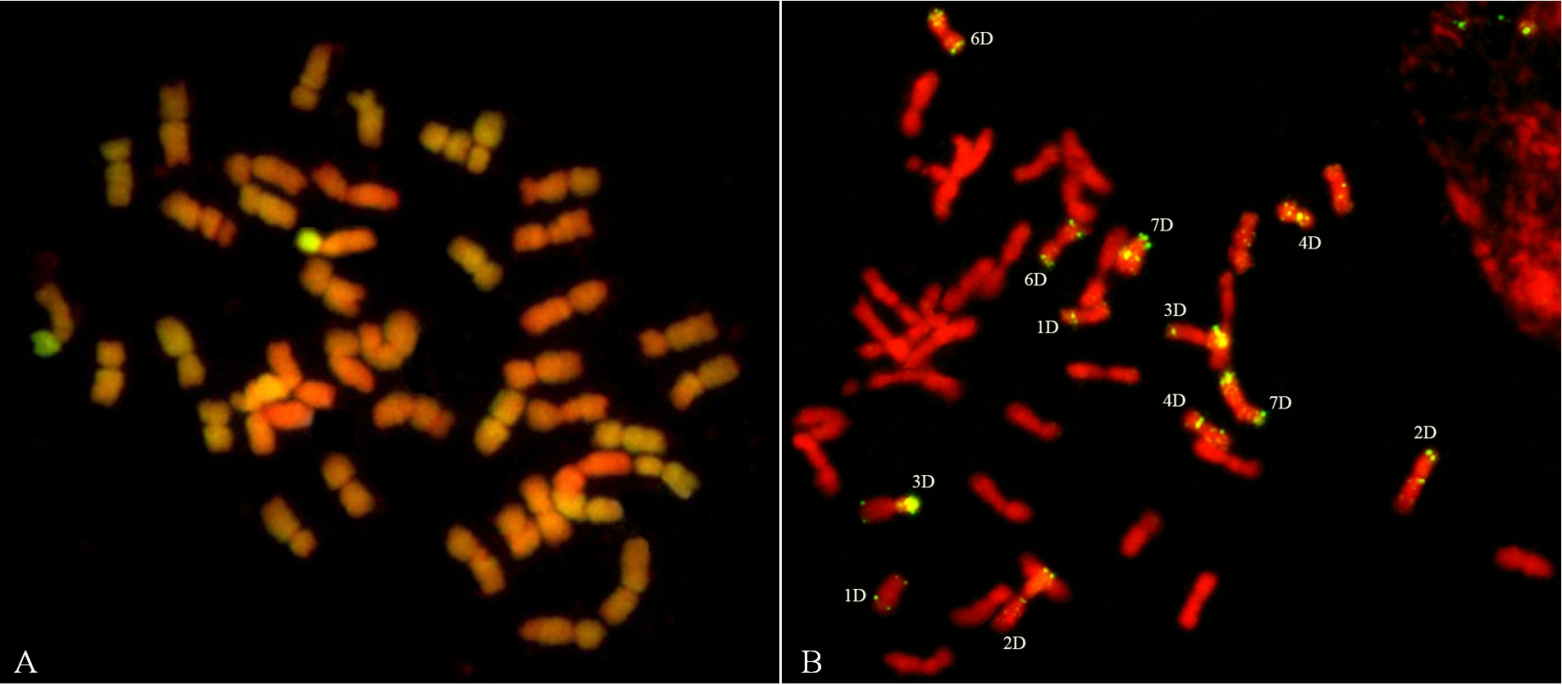
**Genomic *in situ* hybridization (GISH) (a) and fluorescence *in situ* hybridization (FISH) analysis (b) of mitotic chromosome.** The probes used for GISH was *A*. *elongatum* genomic DNA, and for FISH was pAs1.

GISH was used to detect alien genetic materials in 11-20-1. Using *A*. *elongatum* genomic DNA as a probe and sheared CS DNA as a blocker, GISH in the root tip cell of 11-20-1 showed that a pair of chromosome short arms displayed bright green hybridization signals, and this demonstrated that the short arms of a pair of wheat chromosomes were replaced by a pair of *A*. *elongatum* chromosome arms (Fig 2A). Such indicated that 11-20-1 was a genetically stable wheat-*A*. *elongatum* line involving a chromosome translocation.

FISH using wheat D chromosome specific sequence pAs1 as a probe and sheared CS DNA as a blocker was used to discriminate constitution of chromosomes in 11-20-1. The sequenced FISH revealed that 12 D-chromosomes gave rise to clear hybridization signals, only wheat chromosome 5D was absent (Fig 2B). We deduced that the chromosome arms 5DS of wheat were replaced by a pair of *A*. *elongatum* chromosome arms.

### Molecular marker analysis

**Fig 3:**
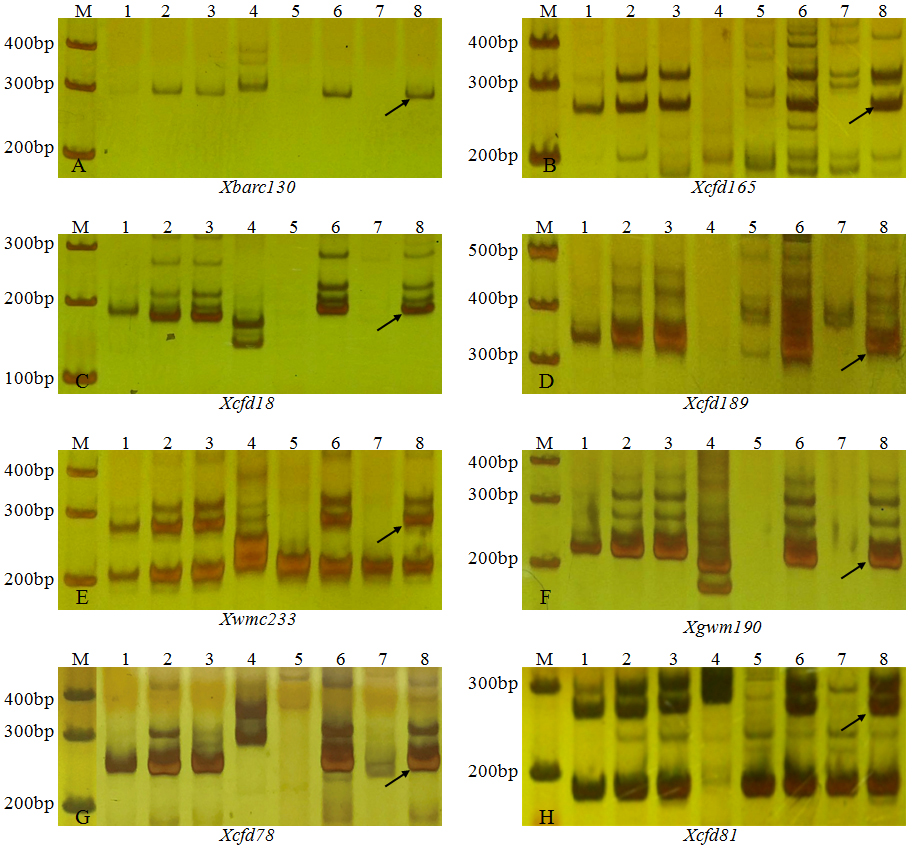
PCR amplification using SSR markers specific for chromosome arms 5DS. M DL100, 1 Lankaozaizaoba, 2 Keyu 818, 3 BainongAK 58, 4 *A*. *elongatum*, 5 11-20-1, 6 Chinese Spring, 7 CSN5DT5A, 8 CSN5AT5D. The arrow indicates a specific band of chromosome arms 5DS.

**Fig 4:**
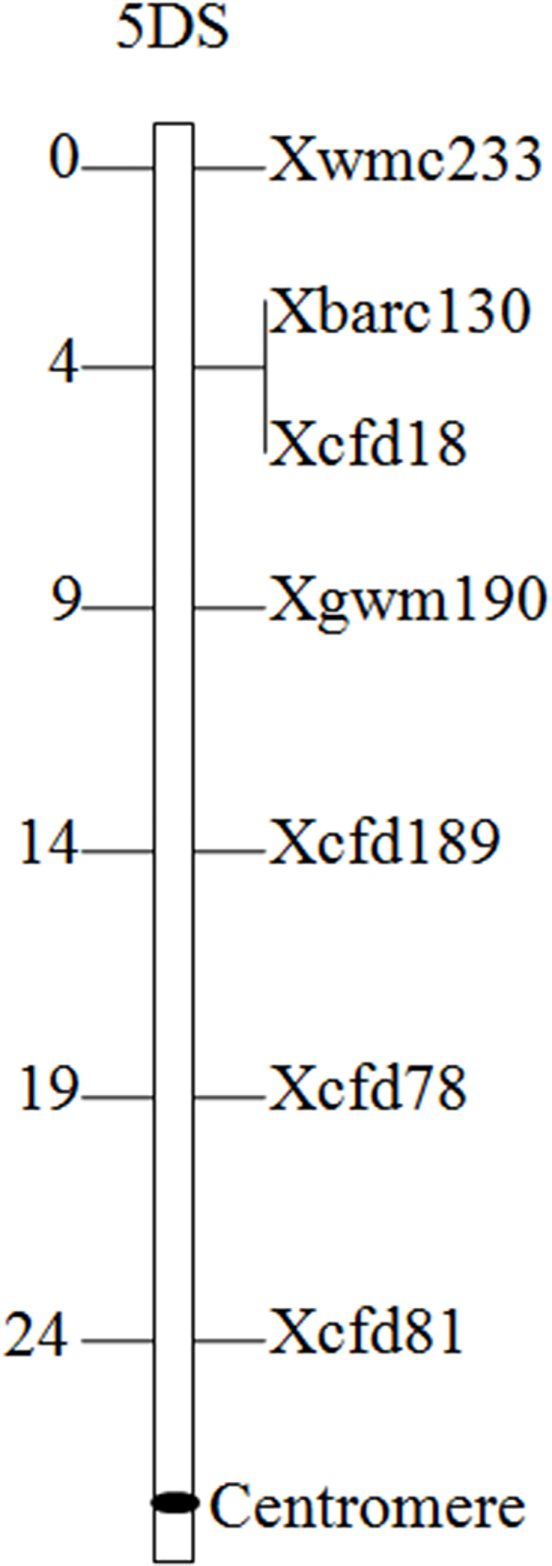
Location of SSR markers specific for chromosome arms 5DS according to Somers et al. [24].

To test the result from GISH/FISH, a total of 560 pairs of microsatellite primers located on different wheat chromosomes were used to analyze the line 11-20-1. Of these SSR markers tested, eight (i.e. Xwmc233, Xbarc130, Xcfd18, Xgwm190, Xcfd189, Xcfd78, Xcfd81 and Xcfd165), known to be on the short arms of wheat chromosome 5D, produced the diagnostic fragments for chromosome arms 5DS in wheat cv. Lankaoaizaoba, Keyu 818, BainongAK 58, CS and CSN5AT5D, but absent in 11-20-1 and CSN5DT5A (Fig 3). Of the 8 markers specific for chromosome arms 5DS, seven were mapped and evenly distributed on the genetic map from Somers et al. [24] (Fig 4). The specific bands of SSR markers on other chromosomes and the long arms of wheat chromosome 5D were present in 11-20-1. These results indicated that the missing chromosome arms were 5DS in 11-20-1, which was in agreement with the results obtained through FISH and GISH analysis.

**Fig 5:**
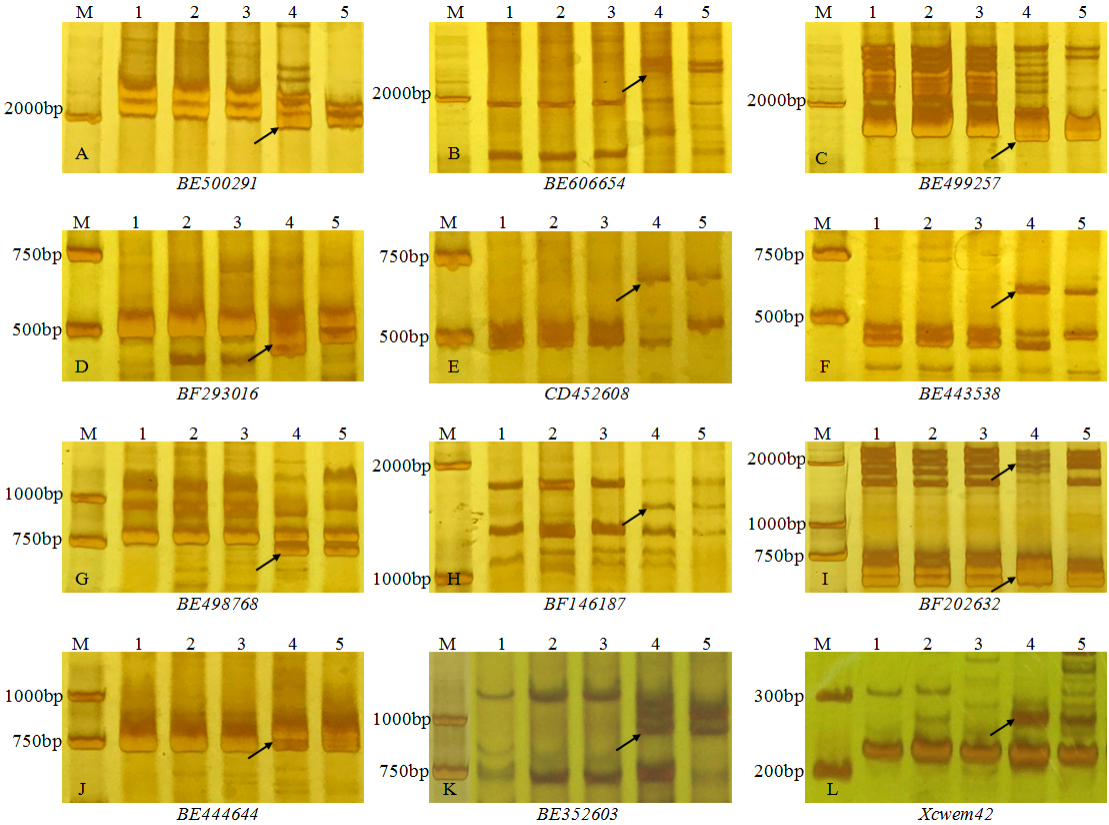
PCR amplification using EST-SSR and EST-STS markers. The arrow indicates an *A*. *elongatum* specific band. M DL100 in the EST-SSR primer Xcwem42 and DL2000 in the EST-STS primer**s**, 1 Lankaozaizaoba, 2 Keyu 818, 3 BainongAK 58, 4 *A*. *elongatum*, 5 11-20-1.

To further determine the homoeologous group relationships of the alien chromosome arms, EST-SSR, EST-STS and PLUG markers distributed on seven homologous groups were used to analyze the line 11-20-1. All PLUG markers used in this study did not amplify specific alleles of *A*. *elongatum* in 11-20-1. One EST-SSR and 11 EST-STS markers (Fig 5), i.e. Xcwem42, BE352603, BE443538, BE444644, BE498768, BE499257, BE500291, BE606654, CD452608, BF146187, BF202632, and BF293016 (Table 1), which were located on 5AS, 5BS and 5DS of wheat chromosomes, amplified fragments specific for *A*. *elongatum* in 11-20-1 but not in the three wheat parent cv. Lankaoaizaoba, Keyu 818 and BainongAK 58. It was suggested that these markers could be used as specific markers of *A*. *elongatum* chromosomes in 11-20-1, and the *A*. *elongatum* chromosome arms that were introduced into 11-20-1 were homoeologous with the fifth chromosome group in wheat. Therefore, it was deduced that the translocated *A*. *elongatum* chromosome arms were 5AgS and 11-20-1 was a wheat-*A*. *elongatum* T5DL∙5AgS translocation line.

**Table 1:** EST-SSR and EST-STS markers on the fifth homoeologous group of wheat applied to linkage analysis of 11-20-1.

Marker	Primer (5’-3’)^a^	Location	Expected fragment size (bp)^b^	Real estimated fragment size (bp)
Xcwem42	F:ACATCCTGGCGGAGAAGTC R:TGGAGAGGTCCTGGTAGGTC	5AS 5BS 5DS	126	270
BE352603	F:GATGGTTGTTGCCACACTTG R:TCATGCTTTGTGTCTGCCTC	5AS 5BS 5DS	708	960/1050
BE443538	F:TTTTGGATGCAGCAAGACAG R:TCTTTGATCAGCTGGGTGTG	5AS 5BS 5DS	410	600
BE444644	F:AAGCTTGCTGAGCTTTCTGG R:TTGAGGGATGTAGGGCAAAG	5AS 5BS 5DS	664	800
BE498768	F:TCGACGAGGATAGGGACATC R:CAGCGAGTGACAATTCCAGA	5AS 5BS 5DS	863	700
BE499257	F:GGTTTGCGTTTGTGGAGTTT R:GGATGACGCACAGGAGATTT	5AS 5BS 5DS	733	1900
BE500291	F:ATGTGCTGATTCCACGAACA R:TGGGACAAAGTCACGCATAA	5AS 5BS 5DS	1300	1950/2100
BE606654	F:AAAAATGAAGCTGTCCGTGG R:ATTCCATTTCACGATCTCGC	5AS 5BS 5DS	-	2400
CD452608	F:TGATGTCTTGTCGTGGTCGT R:TTTTGGATGCAGCAAGACAG	5AS 5BS 5DS	456	650
BF146187	F:CAAGGTGCAACAGTTCATGG R:GGTCACAGAAATATGCGGCT	5AS 5BS 5DS	777	1600
BF202632	F:TCAGCACATTGCCAGAAGTC R:GTGATGACAGGTCACGTTGG	5AS 5BS 5DS	590	1700
BF293016	F:CGTCCTCAAGTCCCTCCTCT R:GAACAGCTCAGCAGAATACGG	5AS 5BS 5DS	553	470

^a^ The annealing temperature was 60 *°*C for all markers

^b^—represent no data

**Fig 6:**
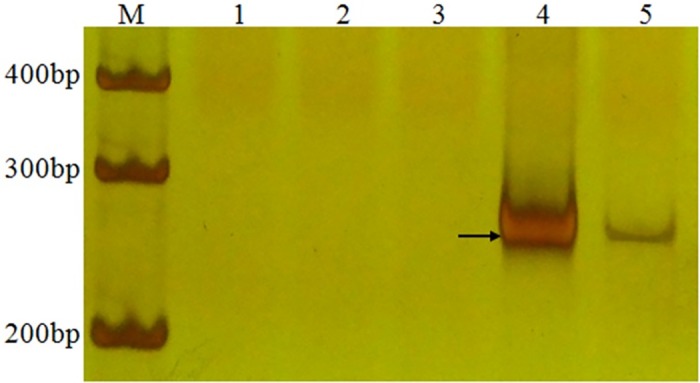
PCR amplification using the marker pLeUCD2. The arrow indicates an *A*. *elongatum* specific band. M DL100, 1 Lankaozaizaoba, 2 Keyu 818, 3 BainongAK 58, 4 *A*. *elongatum*, 5 11-20-1.

The marker, pLeUCD2 specific for E genomic chromatin, was used to identify 11-20-1. A band about 277 bp was amplified in 11-20-1 and *A*. *elongatum*, but did not appear in wheat parent cv. Lankaoaizaoba, Keyu 818 and BainongAK 58 (Fig 6). This indicated that the introgressed *A*. *elongatum* chromosome arms in 11-20-1probably involved E-genome chromatin.

### Disease resistance evaluation

**Fig 7:**
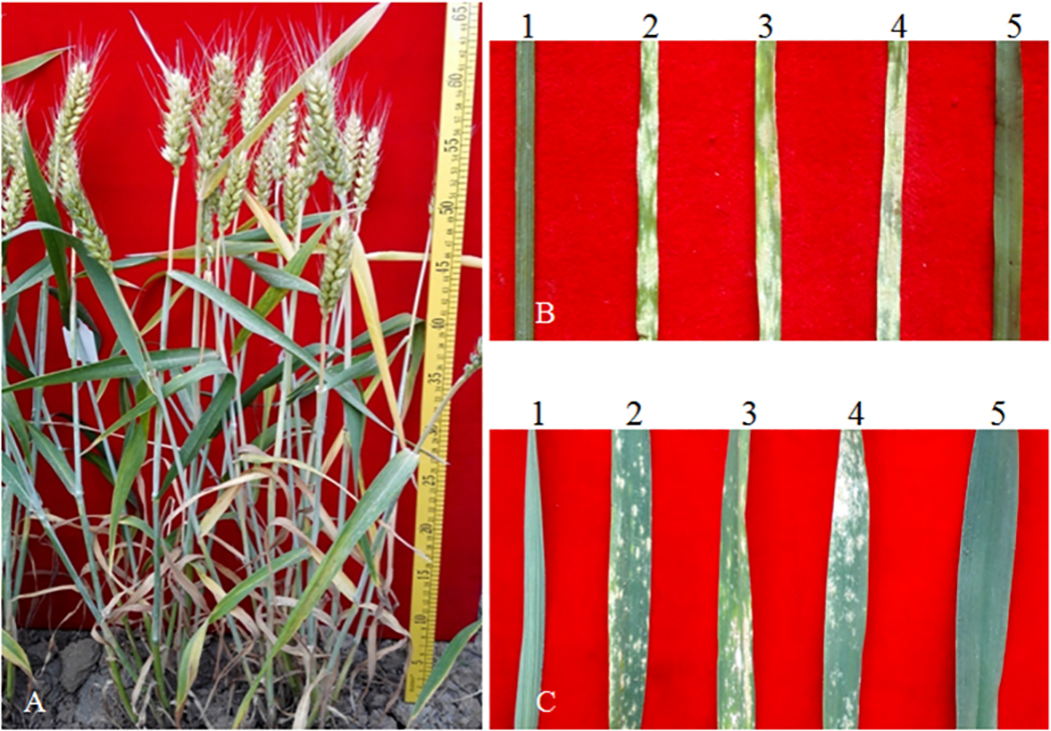
**Plants of 11-20-1 (a) and resistance to powdery mildew at the seedling (b) and adult plant stage (c).** 1 *A*. *elongatum*, 2 Lankaozaizaoba, 3 Keyu 818, 4 BainongAK 58, 5 11-20-1.

The level of disease resistance to powdery mildew was recorded at the seedling and adult plant stage for 11-20-1 and its parents (Table 2). The results showed that wheat parents Lankaoaizaoba, Keyu 818 and BainongAK 58 and control variety CS were high susceptible with IT scores of 4 at the seedling stage, whereas 11-20-1 showed an IT score of 0;. At the adult plant stage, no noticeable symptoms were developed on whole leaves with an IT score of 0 in 11-20-1. In contrast, other three parents and CS were observed to be strongly susceptible with IT scores of 4 (Fig 7). The results indicated that the powdery mildew resistance of 11-20-1 was inherited from *A*. *elongatum*.

**Table 2:** Evaluation of the disease resistance to powdery mildew of the line 11-20-1 and its parents.

Materials	Seeding			Adult plant		
	No. of plant observed^a^	Infection type	Resistance/ susceptibility^b^	No. of plant observed	Infection type	Resistance/ susceptibility
*A*. *elongatum*	-	0	R	-	0	R
Lankaoaizaoba	20	4	S	40	4	S
Keyu 818	20	4	S	40	4	S
BainongAK 58	20	4	S	40	4	S
11-20-1	20	0;	R	40	0	R
Chinese Spring	20	4	S	40	4	S

^a^—represent no data

^b^ R = resistance, S = susceptibility.

**Fig 8:**
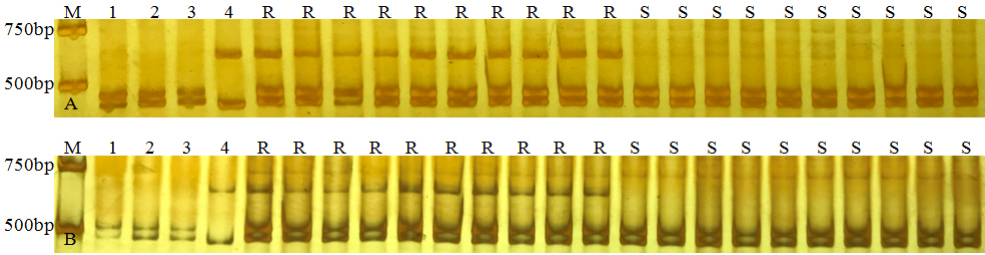
**PCR amplification using two EST-STS markers BE443538 (a) and CD452608 (b) specific for *A*. *elongatum* in 11-20-1.** M DL2000, 1 Lankaozaizaoba, 2 Keyu 818, 3 BainongAK 58, 4 *A*. *elongatum*, R resistant F_2_ plants, S susceptible F_2_ plants.

In order to confirm the powdery mildew resistance of 11-20-1 came from *A*. *elongatum*, 11-20-1 was crossed to susceptible cultivar Yannong 19 to obtain F_1_, and then selfed to get the F_2_ population. The powdery mildew resistance of the F_2_ plants was evaluated under natural conditions at the seedling stage in greenhouses. On the basis of powdery mildew resistance analysis, the genomic DNA of 20 resistant and 20 susceptible plants were further amplified using two specific markers (BE443538 and CD452608) of *A*. *elongatum* chromosomes in 11-20-1. As shown in the Fig 8, the specific bands of *A*. *elongatum* were amplified in the 20 powdery mildew resistant plants, while these bands were absent in the 20 susceptible plants. Such results verified that the *A*. *elongatum* chromosomes were responsible for the powdery mildew resistance.

## Discussion

In this study, the novel wheat line 11-20-1 was produced by crossing with three wheat parents. It was most similar to BainongAK 58 because the variety was crossed two times continuously. BainongAK 58 is a popular variety with many important traits such as lodging resistance, disease resistance and yield potential, and it was bred by Center of Wheat Breeding of Henan Institute of Science and Technology in China. In recent years, BainongAK 58 is always the leading variety in Huang-Huai Rivers Facultative Winter Wheat Region of China, and the total acreage in cultivation has exceeded 20,000,000 hectares sine it was released in 2005 [20]. However, BainongAK 58 was susceptible to powdery mildew. The line 11-20-1 reported in this paper with a valuable background of wheat cultivar BainongAK 58 was not only resistant to powdery mildew, but also stable in agronomic performance. This suggests that line 11-20-1 would be good material for wheat breeding.

Numerous chromosome fragments containing valuable genes have been successfully translocated into wheat from *A*. *elongatum* using methods of chromosome engineering, whereas reports on the transfer of powdery mildew resistance from *A*. *elongatum* to wheat are rare [25]. In this study, we tested the resistance to powdery mildew in wheat line 11-20-1 and its parents, and found that 11-20-1 was almost immune at the seedling and adult plant stage, but three wheat parents (Lankaoaizaoba, Keyu 818 and BainongAK 58) were susceptible to powdery mildew. Both resistance observation and molecular marker analyses using two specific markers (BE443538 and CD452608) of *A*. *elongatum* in the F_2_ population from 11-20-1/Yannong 19 further indicated that the powdery mildew resistance in 11-20-1 came from *A*. *elongatum*. Therefore, the 5AgS chromosome arms of *A*. *elongatum* possibly contained new powdery mildew genes.

To date, four powdery mildew resistance loci/genes have been located on chromosome 5D, i.e. *Pm2* [26], *Pm34* [27], *Pm35* [28], and *PmD57-5D* [29]. *Pm34* and *Pm35* were assigned to chromosome arms 5DL and originated from *Aegilops tauschii*. *Pm2* was mapped to chromosome arms 5DS and found firstly in both hexaploid wheat (Ulka and CI 12632) and *Aegilops tauschii* [26,30]. *PmD57-5D*, which was considered to be *Pm2*, was located on 5DS in a common wheat germplasm D57 [29]. None of these genes is related to *A*. *elongatum*. In addition, in the present study, none of the three markers, Xcfd81, Xgwml90 and Xcfdl8 linked to *Pm2* [30], amplified the 257 bp allele that is diagnostic for the presence of the gene *Pm2* (Fig 3C, 3F and 3H). Recently, Zhan et al. [31] and He et al. [32] identified new powdery mildew resistance genes *Pm51* and *PmSn0224* in wheat-*Th*. *ponticum* introgression lines, respectively, whereas the two genes were localized on chromosome 2BL and 2A. Ji et al. [33] reported that they found a powdery mildew resistant line with introgression of *A*. *elongatum* chromatin. But the material they found was an additional line; it must still be crossed with other wheat genotypes to produce translocation lines for use. Therefore, the translocation germplasm 11-20-1 in the present study may provide a new source of resistance from *A*. *elongatum* and it can be used easily for wheat resistant breeding in future breeding programs.

It has been reported that most spontaneous wheat-*Thinopyrum* translocations and substitutions take place in the D genome of wheat, and some in the A genome and rarely in the B genome [34–36]. For example, Chen et al. [9] identified a novel semi-dwarf line 31505–1, where the introgressed *Th*. *ponticum* segment was located on 2DL. The powdery mildew resistance gene *Pm43* introgressed from *Th*. *intermedium* was also located on 2DL [37]. Zhang et al. [38] localized the Yellow dwarf disease resistant gene of *Th*. *intermedium* to 7DL. The blue-grained wheat lines, Blue Dark and Blue 58 from *Th*. *ponticum*, belonged to 4Ag (4D) disomic substitution lines [39]. Wang et al. [40] localized six small chromosome segments of *A*. *elongatum* in line II-1-3 and found that three of six hybridization signals were positioned on D-genome chromosomes. In this study, in combination with GISH, FISH and SSR analyses, a pair of *A*. *elongatum* chromosome arms were introduced to the line 11-20-1. The translocation was produced spontaneously from the progeny of a wheat-*A*. *elongatum* cross. These results are in agreement with a previous report by Liu et al. [41], who suggested that the reason that the D-genome chromosomes were involved in translocations or substitutions most frequently is possibly because the *Thinopyrum* genome shares higher homology with the D than with the A or B genomes.

SSR markers mapped on the specific sites of wheat chromosomes are used widely for identifying the substituted wheat chromosomes or segment in wheat-alien introgressions in various relatives of wheat, such as *Leymus mollis* [42], *Agropyron cristatum* [43], *Triticum timopheevii* [44], *Secale cereal* [45], *Thinopyrum intermedium* [46], *Agropyron elongatum* [40] and *Aegilops umbellulata* [47]. In our study, eight SSR markers, which did not amplify 5DS-specific bands, were able to successfully identify the absence of wheat chromosome arms 5DS in 11-20-1, and this result was further supported by the FISH analysis.

Recently, EST-PCR markers, which were developed from a conserved coding region that showed high levels of collinearity among the cereal genomes, have been used extensively as powerful techniques for determining the homoeologous relationships of alien chromosomes and tracking alien chromosomes in several species, including *Secale cereal* [48], *Leymus mollis* [15], *Thinopyrum ponticum* [49], *Psathyrostachys huashanica* [50], *Agropyron cristatum* [43] and *Dasypyrum villosum* [51]. In this study, we used a group of EST-PCR markers to assign the linkage group of introduced *A*. *elongatum* chromosomes in 11-20-1. One EST-SSR and 11 EST-STS multiple-loci markers, which were mapped on 5AS, 5BS and 5DS of wheat chromosomes, amplified specific alleles of *A*. *elongatum* in 11-20-1, indicating that the introduced *A*. *elongatum* chromosome arms belonged to the fifth homoeologous group. This finding was in agreement with previous reports [52,53], who suggested that natural substitutions usually involve homoeologous chromosomes, and the alien homoeologous chromosome pair compensates for the loss of wheat chromosomes. Compared with the *in situ* hybridization analyses, these specific markers could identify the translocation lines faster and more effectively, which will be very useful to rapidly identify and trace the translocated fragments in the progenies of translocation lines.

As a complex decaploid species, the genomic composition of *A*. *elongatum* has not been completely clarified. Zhang et al. [54] proposed that E and St were two basic genomes of *A*. *elongatum*, whereas Chen et al. [55] suggested that the chromosome composition in *A*. *elongatum* consists of either the E or J genome, but does not include the St genome. In our experiment, the PCR products with primers specific for the E genome of *A*. *elongatum* were present in 11-20-1, implying that the line 11-20-1 contained E chromatin or chromosome segments of *A*. *elongatum*. Likewise, the specific fragments for the E genome of *A*. *elongatum* were successfully amplified using the same marker in some wheat-*Thinopyrum* progenies by Li et al. [22].

Compared with substitution or addition lines, translocations are preferred and can be used directly in breeding programs. Both radiation treatment and induced homoeologous recombination have been widely used to produce translocation [34]. Recently, inducing chromosome translocation by ethylmethylsulfone (EMS) was reported in *Psathyrostachys huashanica* [56]. In order to improve the utilizability of 11-20-1 in wheat breeding, we are now developing new translocation lines containing smaller alien segments than complete chromosome arms 5AgS by the ^60^Co gamma ray and ethylmethylsulfone treatment, and the *ph1b*-induced homoeologous recombination method. These newly created translocation lines will provide novel germplasms and valuable materials for applied research and resistant breeding in wheat.

In summary, we developed a new wheat-*A*. *elongatum* T5DL∙5AgS translocation line 11-20-1 that was produced spontaneously from the progeny of a wheat-*A*. *elongatum* cross. The 11-20-1 line was almost immune to the isolates of powdery mildew at the seedling and adult plant stages, and analyses revealed that the *A*. *elongatum* chromosome arms 5AgS possessed powdery mildew resistances gene(s). This new line could be further developed as an important parent material in wheat resistance breeding programs.
